# The time since last menstrual period is important as a clinical predictor for non-steroidal aromatase inhibitor-related arthralgia

**DOI:** 10.1186/1471-2407-11-436

**Published:** 2011-10-10

**Authors:** Miyuki Kanematsu, Masami Morimoto, Junko Honda, Taeko Nagao, Misako Nakagawa, Masako Takahashi, Akira Tangoku, Mitsunori Sasa

**Affiliations:** 1 Department of Surgery, Higashitokushima National Hospital, 1-1, Ohmukai-kita, Ootera, Itano, Tokushima, 779-0193, Japan; 2 Department of Oncological and Regenerative Surgery, Institute of Health Biosciences, The University of Tokushima, 3-18-15, Kuramoto-Cho, Tokushima, 770-8509, Japan; 3 Department of Surgery, Tokushima Breast Care Clinic, 4-7-7, Nakashimada-Cho, Tokushima, 770-0052, Japan

## Abstract

**Background:**

The clinical predictors of aromatase inhibitor-related arthralgia (AIA), a drug-related adverse reaction of aromatase inhibitors (AIs), remain unclear.

**Methods:**

AIA was prospectively surveyed every 4 months in 328 postmenopausal breast cancer patients administered a non-steroidal AI (anastrozole). Various clinicopathological parameters were recorded and analyzed (chi-square test, Fisher's exact test and logistic regression analysis).

**Results:**

The mean observation period was 39.9 months. AIA manifested in 114 patients (34.8%), with peaks of onset at 4 (33.7%) and 8 months (11.4%) after starting AI administration. Some cases manifested even after 13 months. AIA tended to occur in younger patients (incidences of 46.3%, 37.4% and 28.0% for ages of < 55, 55-65 and > 65 years, respectively (p = 0.063)) and decreased significantly with the age at menarche (53.3%, 35.3% and 15.4% for < 12, 12-15 and > 15 years, respectively (p = 0.036)). The incidences were 45.1%, 46.3 and 25.1% for the time since the last menstrual period (LMP) < 5 years, 5-10 years and > 10 years, being significantly lower at > 10 years (p < 0.001). In logistic regression analysis, the AIA incidence was significantly lower in the time since LMP > 10-year group versus the < 5-year group (odds ratio 0.44, p = 0.002), but the age at menarche showed no association. AIA manifested significantly earlier (≤ 6 months) as the time since LMP became shorter (< 5 years).

**Conclusion:**

AIA tends to manifest early after starting AI, but some cases show delayed onset. The incidence was significantly lower in patients with a duration of > 10 years since LMP. When the time since LMP was short, the onset of AIA was significantly earlier after starting AI administration.

## Background

Aromatase inhibitors (AI) have shown efficacy that is superior to that of tamoxifen (TAM) in large-scale randomized clinical studies, and they are now extensively used as adjuvant therapy for patients with endocrine-responsive, postmenopausal breast cancer [1,2]. However, aromatase inhibitor-related arthralgia (AIA) is a problematic drug-related adverse reaction of AIs. The incidence of AIA has been reported to be 20%-50% in AI-administered patients [3-5] and, although the number of cases is small, it is sometimes necessary to discontinue AI administration [6-11]. AIA manifests most commonly during the first 3-6 months of AI administration, and it was reported that its occurrence is significantly higher when the time since the last menstrual period (LMP) is short [12]. We have reported similar results [13]. It is thought that a decrease in serum E2 is involved in the onset of AIA [14-20]. We also reported that a decrease in E2 is indirectly involved in AIA manifestation [13], but the details of the clinical predictors have never been elucidated. Here, with the objective of identifying those clinical predictors, we report the findings of our surveillance of AIA in a prospective trial of patients being administered an AI.

## Methods

This study enrolled a total of 390 postmenopausal breast cancer patients who were administered an AI between January 2005 and October 2010 at Tokushima Breast Clinic. The toxicity profiles of steroidal and non-steroidal aromatase inhibitors are known to differ [11], and for that reason we orally administered a non-steroidal AI, anastrozole, at 1 mg/day. The following patient data were recorded at the time of enrollment of each patient: age, age at menarche, number of child deliveries, BMI (the BMI cut-off values were as reported previously [12]), the presence/absence of arthralgia prior to AI administration, the time since LMP, the presence/absence of therapy for breast cancer prior to AI administration (excluding hormone replacement treatment), Stage, the presence/absence of axillary node metastasis, and estrogen receptor status. Surveillance for AIA was performed prior to AI administration and, in principle, at 4-month intervals following the start of AI administration. AIA was defined as new manifestation of joint symptoms (pain or stiffness) following the AI administration or exacerbation of existing joint symptoms following the AI administration. The assessment of AIA was performed as patient-reported outcomes in accordance with the Common Terminology Criteria for Adverse Events (CTCAG) Version 4.0 [21]. In the case of patients with joint symptoms prior to the start of AI administration, the grade of the symptoms at the time of exacerbation was assessed, and the pretreatment symptoms were not taken into consideration. In the end, a total of 328 patients were confirmed to have continuously ingested the AI drug for at least 8 months after the start of administration and had undergone sufficient clinical surveillance for inclusion in this study. The presence/absence of AIA and the time of onset of AIA were compared with the clinicopathological findings. Statistical analysis was performed using the chi-square test and Fisher's exact test and univariate analysis and multivariate analysis were performed using logistic regression analysis. Variables that were not significant at the 0.20 level in the bivariate analyses were not included. A p value of < 0.05 was defined as representing a statistically significant difference in the chi-square test and Fisher's exact test, and < 0.01 indicated significance in multivariate analysis [12,14]. The design of this study was approved by the Ethics Committees of The Institute of Medical Science, The University of Tokyo (trial registration number: 19-11-1211), and The University of Tokushima. Prior informed consent was obtained in writing from each of the enrolled patients.

## Results

### Incidence and Time of Onset of AIA

**Figure 1:**
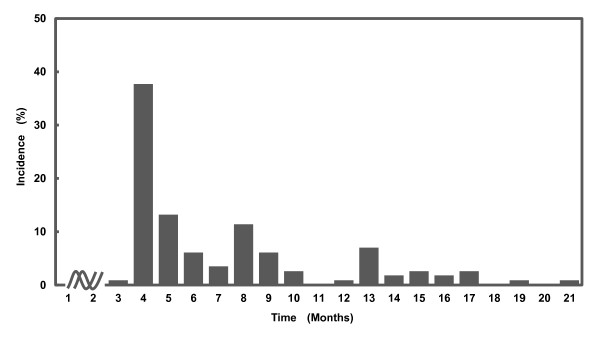
**The time of onset of AIA showed peaks at 4 (33.7%) and 8 months (11.4%) after starting AI administration, and there was onset in a small number of patients even after more than 13 months**.

The mean observation time for the 328 patients was 39.9 months (39.9 ± 19.8) (range 8.9-119.9), and AIA manifested in 114 (34.8%) patients. Next, the time of onset of the AIA following the start of AI administration was investigated. The time of onset showed peaks at 4 months (33.7%) and 8 months (11.4%) after starting AI administration, and there was onset in a small number of patients even after more than 13 months (Figure 1).

### Comparison of Presence/Absence of AIA and the Patient Background Factors

The patient background factors were compared between 214 patients who manifested AIA and 114 patients who did not. The incidence of AIA was 46.3% in patients aged < 55 years, 37.4% in patients aged 55-65 years and 28.0% in patients older than 65. There was a tendency for AIA to manifest more frequently in younger patients (p = 0.063). When stratified on the basis of the age at menarche, i.e., < 12 years, 12-15 years and > 15 years, the incidences of AIA were 53.3%, 35.3% and 15.4%, respectively, and AIA manifested significantly more frequently as the age at menarche became lower (p = 0.036). The incidence of AIA was 31.8% in patients with a BMI of < 25 and 40.7% in patients with a BMI of 25-30; there was no correlation between the BMI and manifestation of AIA. The time since LMP was stratified into < 5 years, 5-10 years and > 10 years, and the incidences of AIA were 45.1%, 46.3% and 25.1%, respectively; the incidence was significantly lower in the patients with time since LMP > 10 years (p < 0.001). With regard to the presence/absence of treatment prior to the start of the AI administration, stratification showed AIA incidences of 39.8% and 31.3% in the presence and absence of prior therapy, respectively. Although the AIA incidence was higher in the patients who had undergone prior therapy, the difference was not statistically significant. In addition, there were no differences in the incidence of AIA as a function of the number of child deliveries, stage, presence/absence of lymph node metastasis, presence/absence of previous hormonal therapy or whether or not a taxane was included in the chemotherapy regimen (Table 1). Next, logistic regression analysis was performed in relation to the age at menarche and the time LMP, which had shown significant differences in the incidence of AIA. The results showed that, when the time since LMP < 5 years was used as the reference, the incidence of AIA in the case of time since LMP > 10 years was significantly lower, with an odds ratio of 0.44 (p = 0.002). On the other hand, no statistically significant difference was found in regard to the age at menarche (Table 2).

**Table 1:** Comparison of Presence/Absence of AIA and the Patient Background Factors

Background		w/o AIA(%)	with AIA(%)	P-value
		n = 214	n = 114	
Age (yrs)	< 55	22(53.7)	19(46.3)	NS*
	55-65	97(62.6)	58(37.4)	
	65 <	95(72.0)	37(28.0)	

Age at menarche (yrs)	< 12	7(46.7)	8(53.3)	*P = *0.036*
	15-Dec	183(64.7)	100(35.3)	
	15 <	22(84.6)	4(15.4)	

No. of childbirths	0	27(71.1)	11(28.9)	NS*
	2-Jan	132(63.5)	76(36.5)	
	2 <	53(66.3)	27(33.8)	

Body mass index (kg/m2)	< 25	150(68.2)	70(31.8)	NS*
	25-30	64(59.3)	44(40.7)	

Past history of Arthritis	Yes	48(71.6)	19(28.4)	NS*
	No	166(63.6)	95(36.4)	

Time since LMP (yrs)	< 5	39(54.9)	32(45.1)	*p *< 0.001*
	10-May	44(53.7)	38(46.3)	
	10 <	131(74.9)	44(25.1)	

Pre-treatment (HT and/or CT)	No	134(68.7)	61(31.3)	NS*
	Yes	80(60.2)	53(39.8)	

Pre-treatment (HT)	No	166(64.8)	90(35.2)	NS**
	Yes	48(66.7)	24(33.3)	

With pre-treatment: (Chemo with taxane)	No	17(51.5)	16(48.5)	NS*
	Yes	27(56.3)	21(43.8)	

Stage	0	17(60.7)	11(39.3)	NS**
	I	112(62.9)	66(37.1)	
	II	80(70.8)	33(29.2)	
	III	5(62.5)	3(37.5)	
	IV	0	1	

Metastasis to LNs	Yes	68(63.6)	39(36.4)	NS*
	No	118(65.9)	61(34.1)	
	Nd	28	14	

ER	+	204(64.4)	113(35.6)	NS**
	-	10(90.9)	1(9.1)	

AIA: aromatase inhibitor related arthralgia; LMP: last menstrual period;

HT: hormone therapy; CT: chemotherapy; Nd: not detected; ER: estrogen receptor;

w/o: without;

*:Chi-square test; **:Fisher's exact test.

**Table 2:** Comparison of Presence/Absence of AIA and the Patient Background Factors

Background	Univariate	Multivariate	*P-*value
	Odds ratio(95%CI)	Odds ratio(95%CI)	
Time since LMP(yrs)			*p *= 0.002*
< 5	Reference	Reference	
10-May	1.08(0.57-2.04)	1.10(0.58-2.09)	
10 <	0.39(0.22-0.71)	0.44(0.24-0.80)	

Age at menarche(yrs)			*p *= 0.24*
< 12	Reference	Reference	
15-Dec	0.48(0.17-1.36)	0.60(0.21-1.76)	
15 <	0.16(0.04-0.69)	0.29(0.06-1.33)	

AIA: aromatase inhibitor related arthralgia; LMP: last menstrual period;

*: logistic regression analysis.

### Comparison of the Time of Onset of AIA and the Patient Background Factors

The time of onset of AIA and the patient background factors were investigated for associations in the patients who manifested AIA. Only the time since LMP showed a clear correlation with the onset of AIA. The time of onset of AIA was divided into 2 groups (≤ 6 months and > 6 months) or 3 groups (≤ 6 months, 7-12 months and > 12 months). AIA was more likely to manifest early after the start of AI administration (≤ 6 months) as the time since LMP became shorter (< 5 years) (Table 3).

**Table 3:** Comparison of the Time of Onset of AIA and the Time Since LMP

	Onset (months)			Onset (months)			
Background	≦6	6 <	P-value	≦6	12-Jul	13≦	P-value
	n = 66	n = 48		n = 66	n = 28	n = 20	
Time since LMP (yrs)			*p *= 0.036*				*p *= 0.032**
< 5	24	8		24	7	1	
10-May	22	16		22	7	9	
10 <	20	24		20	14	10	

AIA: aromatase inhibitor related arthralgia; LMP: last menstrual period;

*Chi-square test; **Fisher's exact test.

## Discussion

The incidence of AIA in this surveillance was 34.8%. With regard to the time of onset of AIA, it had been thought that most cases of AIA manifest within the first 6 months of administration of an AI [12]. However, in this investigation we found that the time of onset showed two peaks, at 4 months and 8 months after starting AI administration, and it was elucidated that some cases manifested even after 13 months of treatment. Such delayed onset was also reported in the ATAC trial [3], and we thus confirmed the delayed onset of AIA.

As factors involved in the onset of AIA, it has been said that most cases develop in patients in whom the time since the LMP is within 5 years and in the 50-59 year-old age bracket [12,22,23]. Our univariate analysis found that AIA tended to increase in incidence in the younger patients, while its incidence was significantly lower in the patient group with a long time since LMP and significantly higher as the age at menarche decreased. In addition, multivariate analysis indicated that the time since LMP was the only factor that correlated significantly with the onset of AIA. Prior treatment, obesity, etc., have been suggested to be other factors that readily cause AIA, but there is no consensus in his regard [22,23]. Our results found that onset of AIA was more frequent in patients who had been treated previously and had a high BMI, but the differences were not statistically significant.

Our analysis of patient background factors that might be related to the time of onset of AIA indicated that this adverse event was significantly more common when the time since LMP was short. There was a peak of onset of AIA at 4 months after starting administration of the AI, but that is when we started our surveillance. Thus, we were unable to determine more accurately the onset of AI at an earlier time. There have been reports that the most common time of onset was within 3 months after starting AI administration [12], and it can be thought that it is a fact that there is a first peak of manifestation soon after starting administration. It can be thought that patients with a short time since LMP are more susceptible to the effects of a decrease in E2 due to an AI drug. It has been hypothesized that estrogen can modulate spinal and central processing of nociception through opioid-containing neurons in the spinal cord and brain that express estrogen receptors, and hypoestrogenemia may increase pain sensitivity by decreasing the pain threshold [5]. In addition, aromatase is present in the osteoblasts, synovial cells and chondrocytes of articular cartilage, and there are reports that surmise that local decreases in E2 in the bone and joints are involved in AIA [4]. However, we think that these two hypotheses might be involved in AIA when the time since LMP is short.

On the other hand, carpal tunnel syndrome, wrist effusion, tendon sheath enhancement and thickening, and tendosynovial changes were reported in AIA patients in a study that investigated the joint findings by sonography performed at a mean of 12 months following administration of an AI [24] and a study that performed objective examination by magnetic resonance imaging at 6 months following AI administration [25]. Those studies indicated the possibility that organic changes occur in the joints, but it is difficult to believe that they could manifest within a short period of time, and that a certain length of time would be necessary for them to occur.

In our present study, we found that AIA did not manifest only in the early period after starting AI administration, that it also occurred after a certain amount of time had passed and that there was even delayed onset of AIA. The time since LMP was long in many of the patients with delayed onset of AIA, and we surmise that the clinical predictors involved in delayed AIA are different from those in patients with early onset of AIA. It can be thought that, in the future, it will be necessary to clarify the clinical predictors of AIA by carrying out a prospective study that employs E2 monitoring, image assessment using MRI of the joints, etc., and accurate assessment of joint movement [26] during the early period following the start of AI administration.

## Conclusion

AIA is most likely to manifest in the early period after AI administration, but there are also cases of delayed manifestation. It was surmised that the length of the time since LMP is important for elucidating the mechanism of the onset of AIA.

## Abbreviations

AI: aromatase inhibitor; TAM: tamoxifen; AIA: aromatase inhibitor-related arthralgia; LMP: last menstrual period; E2: estradiol; BMI: body mass index; CTCAG: Common Terminology Criteria for Adverse Events.

## Competing interests

The authors declare that they have no competing interests.

## Authors' contributions

MS initiated and co-wrote the paper with MK, MM, JH, TN, MN and MT, and HT took part in the care of patients. AT helped in preparation of the manuscript. All authors read and approved the manuscript.

## Pre-publication history

The pre-publication history for this paper can be accessed here:

http://www.biomedcentral.com/1471-2407/11/436/prepub
